# Supplementary material for: Aggression in Group-Housed Male Mice: A Systematic Review
Source: Animals (Basel). 2022 Dec 30;13(1):143. doi: 10.3390/ani13010143 (PMC9817818; doi:10.3390/ani13010143)
Supplement: Supplementary file 1 [file animals-13-00143-s001.zip › Supplementary Figure S1.pdf]

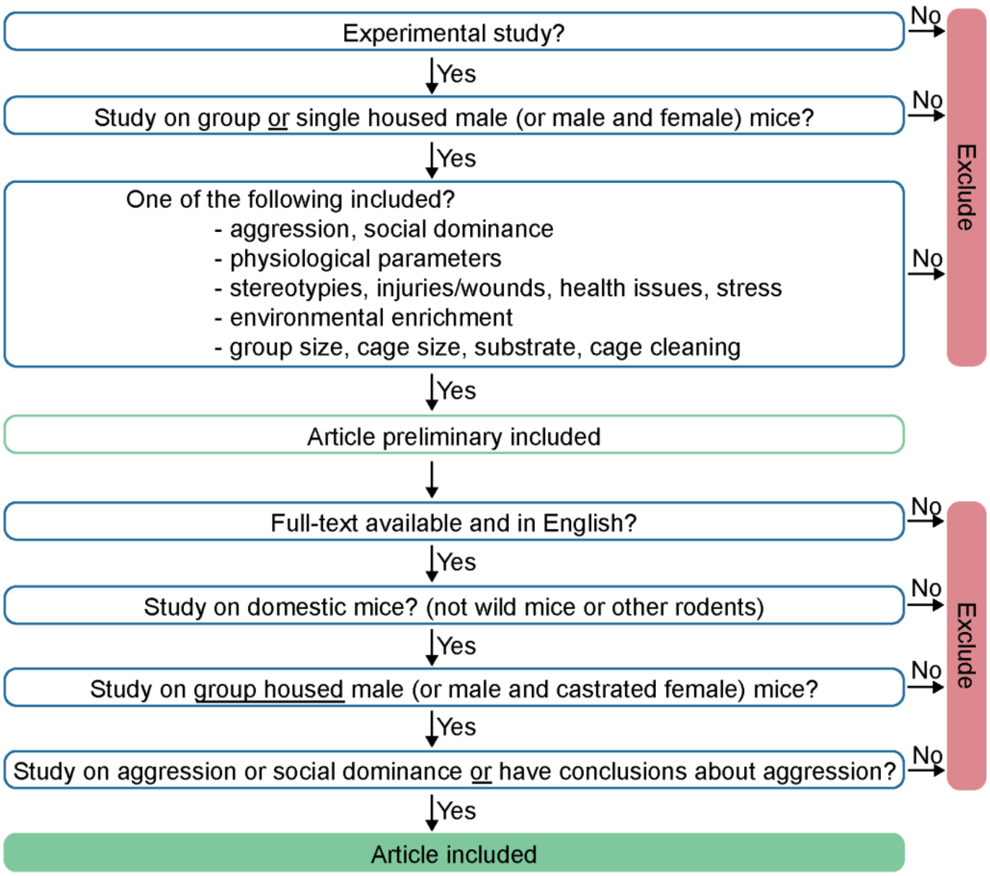

Supplementary Figure S1. Outline of the screening strategy with inclusion and exclusion criteria.
