# Supplementary material for: Aggression in Group-Housed Male Mice: A Systematic Review
Source: Animals (Basel). 2022 Dec 30;13(1):143. doi: 10.3390/ani13010143 (PMC9817818; doi:10.3390/ani13010143)
Supplement: Supplementary file 1 [file animals-13-00143-s001.zip › Supplementary Information Search strings.pdf]

Supplementary Information: Search strings

The initial search strings for Medline was:

1. exp Aggression/
2. Anxiety/
3. ((aggressi* or anxiety or fight* or injur* or stress* or wound*) adj3 (male adj3 (mice or mouse))).ti,ab,kf.
4. (behavio* adj3 (defence or defense or social or submission or territorial)).ti,ab,kf.
5. (social* adj3 (conflict or dominan* or hierarch* or position* or rank* or relationship*)).ti,ab,kf.
6. (territorial adj3 (defence or defense)).ti,ab,kf.
7. or/1-6

8. Housing, Animal/
9. Animal Husbandry/
10. (male adj3 (mice or mouse) adj3 (hous* or husbandry)).ti,ab,kf.
11. ((cage* or cohous* or hous*) adj3 (aggressi* or condition or environment or group* or individual or isolat* or separat* or singl* or social*)).ti,ab,kf.
12. or/8-11

13. exp Mice/
14. Male/
15. 13 and 14
16. (male adj3 (mice or mouse)).ti,ab,kf.
17. or/15-16

18. 7 and 12 and 17
19. limit 18 to english language

The initial search strings for Embase was:

#1 'aggression'/exp
#2 'anxiety'/de
#3 (male NEAR/3 (mice OR mouse) NEAR/3 (aggressi* OR anxiety OR fight* OR injur* OR stress* OR wound*)):ti,ab,kw
#4 (behavio* NEAR/3 (defence OR defense OR social OR submission OR territorial)):ti,ab,kw
#5 (social* NEAR/3 (conflict OR dominan* OR hierarch* OR position* OR

rank* OR relationship*)):ti,ab,kw
#6 (territorial NEAR/3 (defence OR defense)):ti,ab,kw
#7 #1 OR #2 OR #3 OR #4 OR #5 OR #6

#8 'animal housing'/de
#9 'animal husbandry'/de
#10 (male NEAR/3 (mice OR mouse) NEAR/3 (hous* OR husbandry)):ti,ab,kw
#11 ((cage* OR cohous* OR hous*) NEAR/3 (aggressi* OR condition OR environment OR group* OR individual OR isolat* OR separat* OR singl* OR social*)):ti,ab,kw
#12 #8 OR #9 OR #10 OR #11

#13 'mouse'/exp
#14 'male animal'/de
#15 (male NEAR/3 (mice OR mouse)):ti,ab,kw
#16 #13 OR #14 OR #15

#17 #7 AND #12 AND #16 AND [english]/lim

#18 #17 AND ('article'/it OR 'article in press'/it OR 'conference paper'/it OR 'review'/it)

The initial search strings for Web of Science was:

#1 TOPIC: (((aggressi* or anxiety or fight* or injur* or stress* or wound*) NEAR/3 (male NEAR/3 (mice or mouse)))) OR TOPIC: ((behavio* NEAR/3 (defence or defense or social or submission or territorial))) OR TOPIC: ((social* NEAR/3 (conflict or dominan* or hierarch* or position* or rank* or relationship*))) OR TOPIC: ((territorial NEAR/3 (defence or defense)))

#2 TOPIC: ((male NEAR/3 (mice or mouse) NEAR/3 (hous* or husbandry))) OR TOPIC: (((cage* or cohous* or hous*) NEAR/3 (aggressi* or condition or environment or group* or individual or isolat* or separat* or singl* or social*)))

#3 TOPIC: ((male NEAR/3 (mice or mouse)))

#4 #1 AND #2 AND #3 AND LANGUAGES: (ENGLISH)

#5 #4 Refined by: DOCUMENT TYPES: (ARTICLE OR PROCEEDINGS PAPER OR REVIEW)
